# The whole genomic analysis of orf virus strain HN3/12 isolated from Henan province, central China

**DOI:** 10.1186/s12917-017-1178-1

**Published:** 2017-08-18

**Authors:** Huiqin Chen, Wei Li, Zhenzhan Kuang, Daxiang Chen, Xiaoqing Liao, Ming Li, Shuhong Luo, Wenbo Hao

**Affiliations:** 1 Institute of Antibody Engineering, School of Laboratory Medicine and Biotechnology, Southern Medical University, Guangzhou, 510515 People’s Republic of China; 2 Department of Laboratory Medicine, School of Stomatology and Medicine, Foshan University, 5 Hebin Road, Foshan, Guangdong Province 528000 People’s Republic of China; 3 Guangdong Provincial Key Laboratory of Tropical Disease Research, School of Public Health, Southern Medical University, Guangzhou, 510515 People’s Republic of China

**Keywords:** Parapoxvirus, ORFV, HN3/12, Phylogenetic analysis, Genomic sequencing

## Abstract

**Background:**

The Orf virus (ORFV) is the causative agent of orf, a globally-occurring, acute, pustular, contagious disease affecting sheep, goats and humans with a worldwide distribution. Currently, the genomic analysis of four ORFV strains from the Fujian province in southern China and a NA1/11 strain isolated from the Jilin province in northeast China have been reported. However, little is known about the genomic information of ORFV strains from central China.

**Results:**

From a recent outbreak in a sheep herd in the Henan province of central China, a novel ORFV strain (HN3/12) was isolated and cultured in ovine fetal turbinate (OFTu) cells. The strain was identified as HN3/12 and verified by PCR based on the DNA sequences of *011* and *059* genes. The whole genomic sequence of this isolate was determined by Next Generation Sequencing technology. To determine the genetic characteristics of the HN3/12 strain, phylogenetic analysis of the 011 and 059 genes and amino acid sequence alignment of the HN3/12 strain were performed and compared with reference parapoxvirus strains.

**Conclusions:**

The HN3/12 genome is 136,643 bp in length, contains 63.67% G + C and encodes 132 putative genes. Phylogenetic analysis of the 011 and 059 nucleotide sequences showed that this viral strain was similar to the NA1/11 isolate. The homology analysis indicates that HN3/12 has 93% to 98% identity with published ORFV strains at amino acid level. When open reading frames (ORFs) were aligned among the HN3/12 and four Fujian ORFV strains, most of them have identities greater than 90% and only a few less than 60%. The availability of the whole genomic sequence of HN3/12 aids in our understanding of, and provides new insights into, the genetic diversity of ORFV.

**Electronic supplementary material:**

The online version of this article (doi:10.1186/s12917-017-1178-1) contains supplementary material, which is available to authorized users.

## Background

Orf, also known as contagious ecthyma, contagious pustular dermatitis, or scabby mouth, is an acute disease that infects mainly sheep and goats [1–3]. The disease has a worldwide distribution and is characterized by typically self-resolving progress from erythema, via vesicle formation, to pustules and then to scabs [3–6]. It is a zoonotic disease, occasionally causing occupational infections in humans who are in close contact with infected animals [5]. In humans, the common invasion sites of ORFV are the keratinocytes and epithelial cells of the hands and fingers [5, 7]. Although considered a mild disease, orf usually lasts for 3–4 weeks and resolves within 1–2 months. However, mortality rates of up to 93% have been reported in lambs [1, 6, 8, 9]. High mortality rates are generally due to secondary infections or extremely debilitating conditions. Reinfection is commonly observed in sheep [10].

Orf infection has occurred endemically in many countries over the past several years [11]. Since the 1950s, outbreaks of orf have occurred in sheep and goats in more than 20 provinces of China, including the Jilin [6, 9], Hubei [12], Xinjiang and Shaanxi [13], Shandong [14], and Fujian provinces [15, 16]. Although vaccination has been implemented in some regions, morbidity and economic losses remain significant. Attenuated orf virus vaccines can relieve the symptoms of the infection but they are unable to completely prevent the occurrence of orf [17]. Additionally, vaccination is not a common practice in most herds.

The Orf virus (ORFV), the causative agent of orf, is a prototype of the genus Parapoxvirus (PPV)*,* belonging to the family *Poxviridae*, sub-family *Chordopoxvirinae*. The Parapoxvirus genus includes the bovine popular stomatitis virus (BPSV), pseudocowpox virus (PCPV) and parapoxvirus of red deer in New Zealand (PVNZ) [12, 18, 19]. Parapoxviruses can be distinguished from other members of poxvirus genera by their ovoid-shaped virions with relatively small size (about 260 nm in length and 160 nm in width) and the crisscross-pattern tubule-like structure on the particle surface [17].

In spite of the worldwide distribution of ORFV, little information regarding the molecular epidemiology of orf and relatively few molecular characterizations of ORFV strains is available. To date, several complete genomic sequences of ORFV strains isolated from affected sheep or goats have been reported [11, 15, 20–22]. In recent studies, two conserved genes *ORFV011 (B2L)* and *ORFV059 (F1 L)* have been widely used as molecular targets for the phylogenetic analysis of ORFV isolates [6, 23]. Currently, in China, the genomic analysis of four strains (OV-GO, OV-YX, OV-NP and OV-SJ1) from the Fujian province in Southern China [15] and a NA1/11 strain isolated from the Jilin province in Northeast China [22] have been reported. Four Fujian ORFV strain analyses showed gene deletion possibly leads to attenuation of ORFVs, and 47 of the 132 genes can be easily distinguished as originating from sheep or goats. The phylogenetic analysis revealed that the NA1/11 strain was closely related to the Xinjiang and Gansu strains. The nMDS analysis, based on NA1/11 and parapoxvirus reference strains, revealed that geographic locations and animal hosts are likely major factors causing genetic differences among ORFV strains. However, little is known about the genomic information for ORFV viruses from other provinces of China.

Here, we report about an outbreak of orf infection in sheep on a farm located in Wuyang country, Henan province, in the central region of China. The isolated ORFV strain was verified by PCR of the full-length *ORFV011* and *059* genes. Sequence alignment and phylogenetic analysis of *ORFV011*and *059* genes between this strain and other reported stains were carried out. A novel orf virus strain, ORFV-WY-HN (HN3/12), was then successfully isolated and its genomic sequence was determined. The present study provides basic data regarding the new ORFV strain isolated from the north-central region of China, including genomic and phylogenetic analyses.

## Results

### Clinical gross pathological changes

**Fig. 1:**
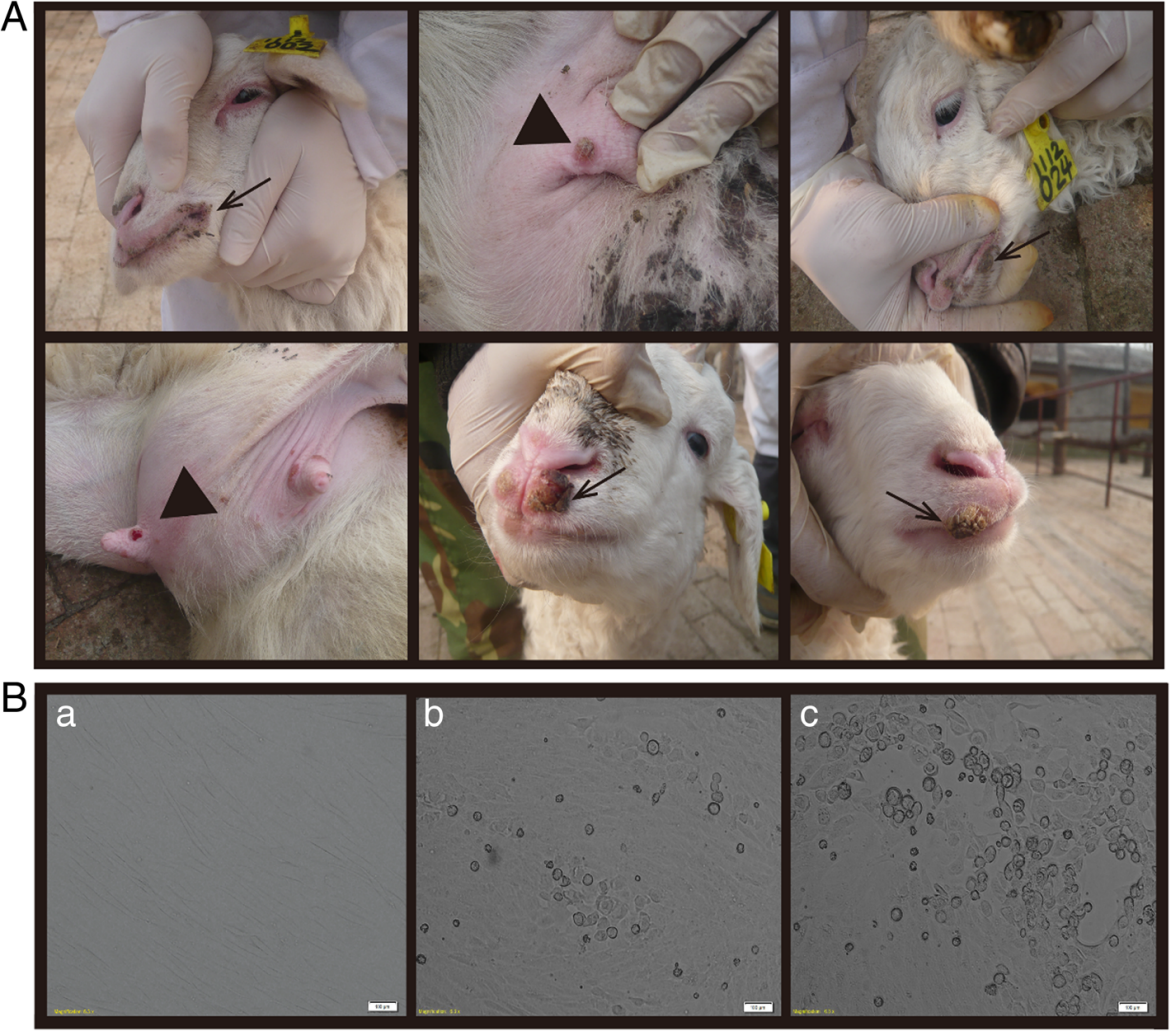
Typical clinical signs of orf virus infection in sheep and OFTu cells. **a** Skin lesions of 6 infected sheep. Typical proliferative lesions on the skin of nostrils (*arrows*), nipple and mouth (*Triangular arrowheads*). **b** Cytopathic effect (CPE) of ovine fetal turbinate (OFTu) cells infected by orf virus strain HN3/12. **a** Uninfected OFTu cells **b** ORFV-HN-WY in OFTu at six days p.i. **c** ORFV-HN-WY in OFTu at eight days p.i. Scale bars = 100 um

Orf, which is endemic in China, has not been effectively controlled due to the lack of an implemented vaccination program. In the sheep herd of Wuyang county, skin lesions displaying clinical symptoms characteristic of orf, such as papules, pustules, and scabs, were recorded in 11 lambs and six ewes. Four lambs and two ewes showed ulcerated or proliferative lesions in the epidermis of the lips, nostrils and nipples when examined (Fig. 1a). For all lambs and ewes, the characteristics of weight loss, anorexia, and proliferative papillomatous nodules ranging from 3 to 5 cm in diameter had appeared. All 17 infected sheep recovered about 30 days after the first clinical signs appeared. The morbidity of the outbreak was 4.25% (17 out of 400) and mortality was 0. No farm staff members were infected from the disease in this outbreak.

### Isolation and culture of virus

All homogenates from the skin lesions were inoculated in OFTu cells. When about 80% cytopathic effect (CPE) was observed, the inoculated cells were passaged three times. Cells with CPE and the medium were harvested and frozen. Healthy OFTu cells were cultured with 1× MEM, as mentioned previously, and inoculated with a filtered virus suspension. Rounding, pyknosis, detachment and eventual degeneration of the OFTu cells infected with virus were observed at 6–8 days in the fourth blind passage (P4) period, corresponding with CPE. The CPE of OFTu cells progressed slowly, leading to grape-like clusters and cell detachment from the monolayer (Fig. 1b). The cells with specific CPE were confirmed by PCR. The isolated virus from the single plaque caused by a single virus was amplified, purified, titrated and preserved at −80 °C. This ORFV strain was successfully isolated and cultured in OFTu cells, and was named ORFV/Henan-C3/2012 (HN3/12).

### PCR amplification

**Fig. 2:**
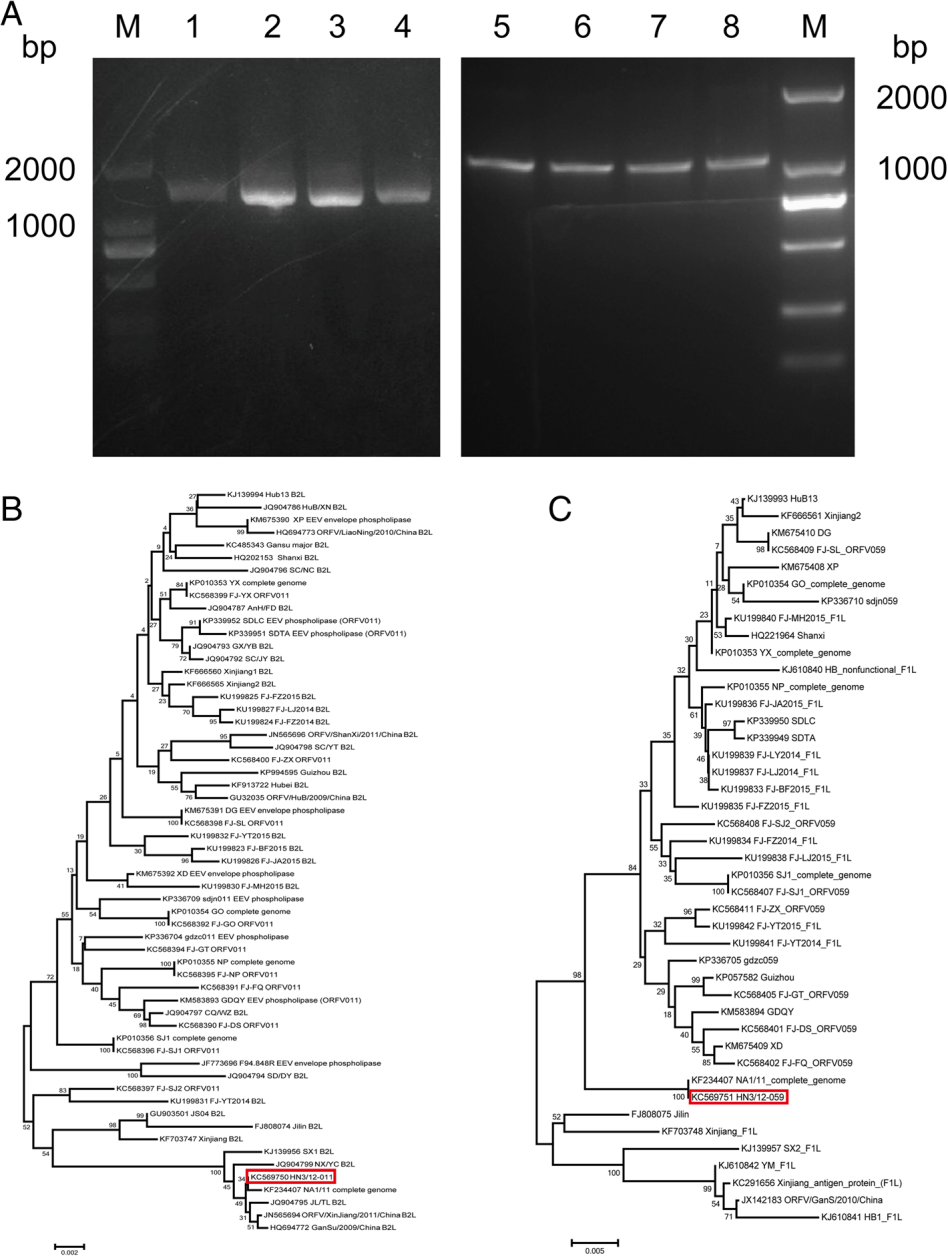
PCR amplification and phylogenetic analyses of ORFV011 (B2L) and ORFV059 (F1 L) of the HN3/12 strain. **a** Amplification of major envelope genes (ORFV 011 and ORFV 059) by PCR. ORFV011 (lane 1, 2, 4) and ORFV059 (lane 5, 6, 8) were amplified from genomic DNA isolated from HN3/12. Lane 3 and 7: DNA extracted from NA1/11 strain as a positive control. Lane M: 2 kb DNA ladder. Phylogenetic analysis of different ORFV strains around the world, based on nucleotide sequences of ORFV011 (**b**) and ORFV059 (**c**). The phylogenetic trees were constructed by the neighbor-joining algorithm using MEGA version 5.2 software. One thousand bootstrap replicates were subjected to nucleotide sequence distance (cut-off value of 50% from 1000 bootstrap replicates). All bootstrap values are displayed above branches. The *scale bar* indicates the branch length. *Red frame*: HN3/12 in this study

To verify whether or not the ORFV had been isolated, two sets of primers for *ORFV011* (1137 bp) and *ORFV059* (1017 bp) were designed. Two conserved genes were amplified from genomic DNA extracted from inoculated cell cultures or purified viral particles. Viral DNA from NA1/11 strain was used as a positive control. The three samples produced distinct positive bands with sizes corresponding to *B2L* (1137 bp) and *F1 L* (1017 bp) (Fig. 2a). PCR products were purified and cloned into a pMD-19 T vector, sequenced in both orientations, and the nucleotide sequence data showed that they were ORFV sequences. *ORFV011* and *059* nucleotide sequences of each sample were identical, indicating that the pathogen of the epidemic disease is the same as on the sheep farm. The *HN3/12–011* and *059* sequences were edited, aligned, and uploaded to GenBank with the following accession numbers: KC569750 (*ORFV011*) and KC569751 (*ORFV059*).

### Phylogenetic analysis of ORFV011 and ORFV059 genes

To determine the genetic relation of the HN3/12 isolate to other ORFV strains, the corresponding nucleotide sequences of *ORFV011* and *ORFV059* genes from different strains were obtained from GenBank, aligned and analyzed using the neighbor-joining method by MEGA 5.2. Bootstrap analysis of *HN3/12-ORFV011* showed that these ORFV strains cluster together into two large branches based on the geographical position. In these ORFV strains, most of the strains from the Fujian province of southern China cluster together, while the Henan strain was closer to the isolates from northern China, such as Henan, Shaanxi, Ningxia, Jilin, Xinjiang and Gansu provinces. The HN3/12 strain is 99% identical with these northern Chinese ORFV strains for nucleotide sequences and 99%–100% for deduced amino acid sequences (Table 1). Among these ORFV strains, the Henan orf virus (HN3/12) is closest to ORFV-NA1/11 isolated from the Jilin province (Fig. 2b). Phylogenetic analysis of *ORFV059* showed that all ORFV strains cluster into two branches. HN3/12 was close to NA1/11, similar to *ORFV011*. However, these two strains cluster into the first branch, in which most ORFVs are from the Fujian province (Fig. 2c).

**Table 1:** The identity of nucleotide and amino acid sequences between the HN3/11 strain and other ORFV strains

Strain and its Geninfo identifier (gi)		ORFV011	
		Nucleotide	Amino acid
KJ1339956	SX1	99	100
KF234407	NA1/11	99	100
JQ904799	NY/YC	99	99
JQ904795	JL/TL	99	99
JN565694	Xinjiang/2011	99	100
HQ694772	Gansu/2009	99	100

### Analysis of genomic sequences of HN3/12

Genomic sequences of ORFV-HN3/12 were assembled into a contiguous sequence of 136,643 bp containing132 genes, with 63.7% G + C content. The left-most nucleotide was arbitrarily designated base 1. Aligned with published reference ORFV strains (OV-GO, OV-YX, OV-NP, OV-SJ1, OV-NZ2, OV-SA00, OV-IA82 and OV-NA1/11) for whole genome sequences, the HN3/12 strain showed the highest homogeneity with NA1/11, having 99% nucleotide identity and 98% amino acid identity. The identities of the HN3/12 strain with other ORFV strains were 97%–98% at the nucleotide level and 93%–97% at the tandem deduced amino acid level (Table 2). Like the other ORFV isolates, the ORFV-HN3/12 genome contains a large central coding region bounded by two identical inverted terminal repeat (ITR) regions. ITRs of ORFV-HN3/12 contain 2794 bp and are the shortest in length, excepting OV-NP strain (2426 bp) (Table 2).

**Table 2:** Summary of complete genomic sequence data of 10 ORFV strains

Isolate	Species of origin	Country of origin	No. predicted genes	Genome size(bp)	ITR size (bp)	Genome G + C (%)	Genbank accession. no.	% ID (nt)	% ID (aa)	References
HN3/12	Sheep	China (HN)	132	136,643	2794	63.7	KY053526	100	100	In this study
NA1/11	Sheep	China (JL)	132	137,080	3020	63.6	KF234407	99	98	Li et al.,2014 [22]
GO	Goat	China (FJ)	132	139,886	3964	63.6	KP010354	97	95	Chi et al.,2015 [15]
YX	Goat	China (FJ)	132	138,231	3446	63.8	KP010353	97	95	Chi et al.,2015 [15]
SJ1	Goat	China (FJ)	129	139,112	4153	63.6	KP010356	97	96	Chi et al.,2015 [15]
NP	Goat	China (FJ)	124	132,111	2426	63.8	KP010355	98	94	Chi et al.,2015 [15]
SA00	Goat	USA	132	139,962	3936	63.4	AY386264	98	93	Delhon et al.,2004 [20]
IA82	Sheep	USA	132	137,241	3092	64.3	AY386263	98	96	Delhon et al.,2004 [20]
NZ2	Sheep	New Zealand	132	137,820	3389	64.3	DQ184476	98	97	Mercer et al.,2006 [21]
D1701	Sheep	Germany	288	134,038			HM133903	97	94	McGuire et al.,2012 [24]

**Fig. 3:**
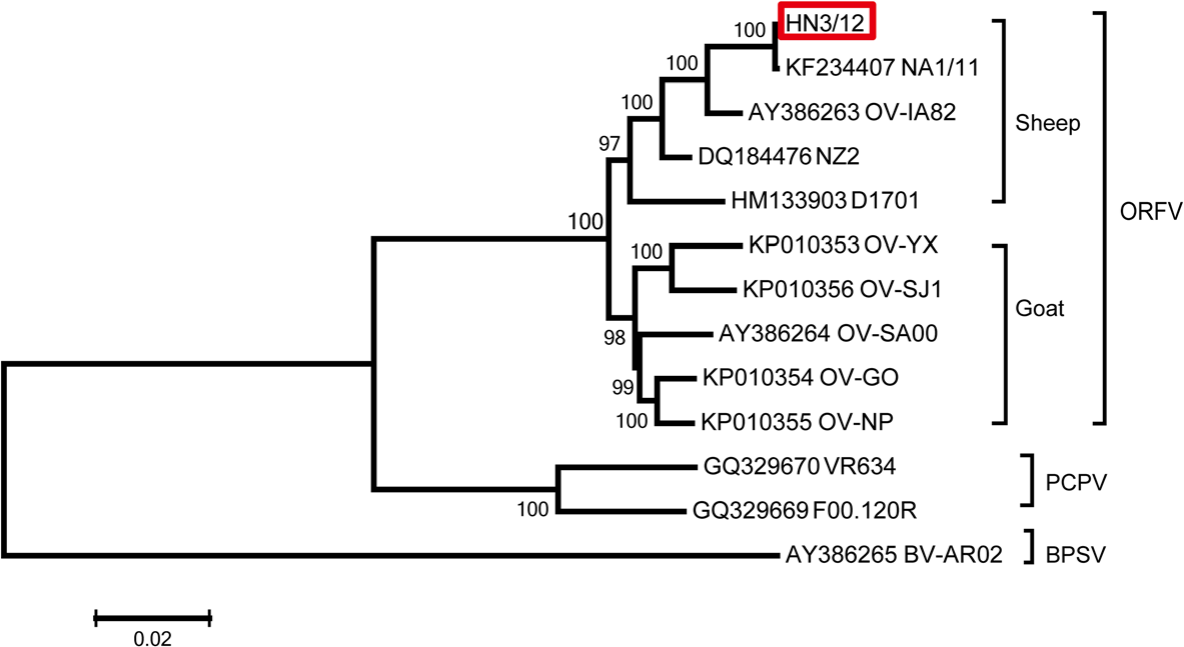
Phylogenetic comparison of PPVs. Genomic nucleotide sequences, including terminal repetitions, were aligned using Clustal W.Phylogenetic trees were generated using the maximum-likelihood algorithm by MEGA 5.2 software. *Numbers* at the branching points indicate the bootstrap support calculated for 1000 replicates (cut-off value is 50%)

Phylogenetic analyses based on the complete genomic sequences of 13 PPV strains revealed that five ORFV strains originating in goats and five stains of sheep origination formed two separate branches with 100% bootstrap support. The ORFV strain from the Henan region showed a close relationship with NA1/11. Among strains originating in sheep, HN3/12 was closer to IA82 than to NZ2 or to D1701. Our analysis also showed that ten ORFVs were more closely related to PCPV than to BPSV (Fig. 3).

### Amino acid sequence alignment in five ORFVs

**Fig. 4:**
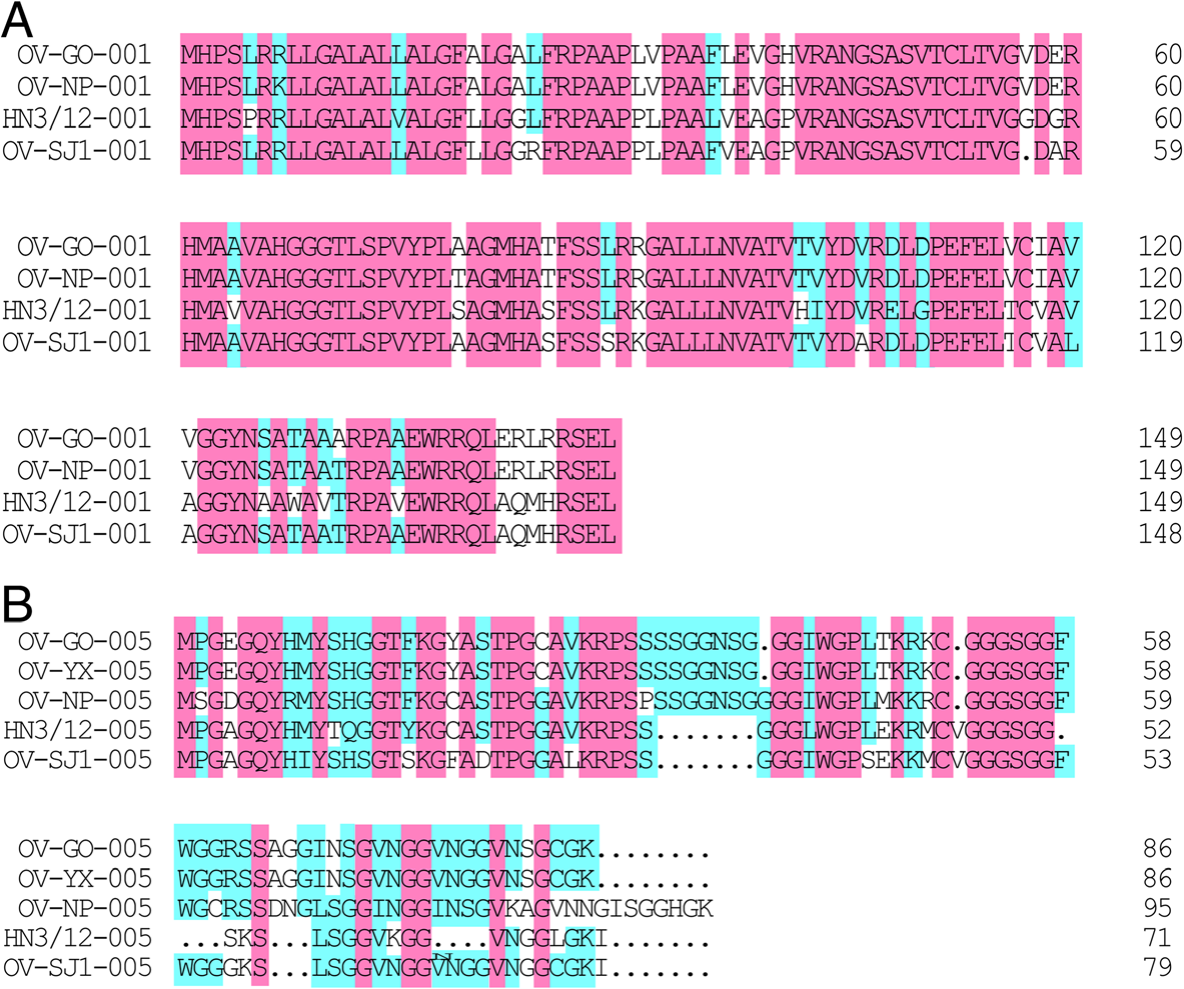
Amino acid sequence alignment of ORF001 and ORF005 coded by five ORFVs. Multiple alignment was performed by Clustal Omega (available at http://www.ebi.ac.uk/Tools/msa/clustalo/) and DNAMAN software. Five ORFV strain accession numbers are shown in Table 2. The *pink box* indicates 100% ID of amino acid sequence among five ORFV strains. The *cyan box* indicates >50% ID of amino acid sequence among five ORFV strains

**Fig. 5:**
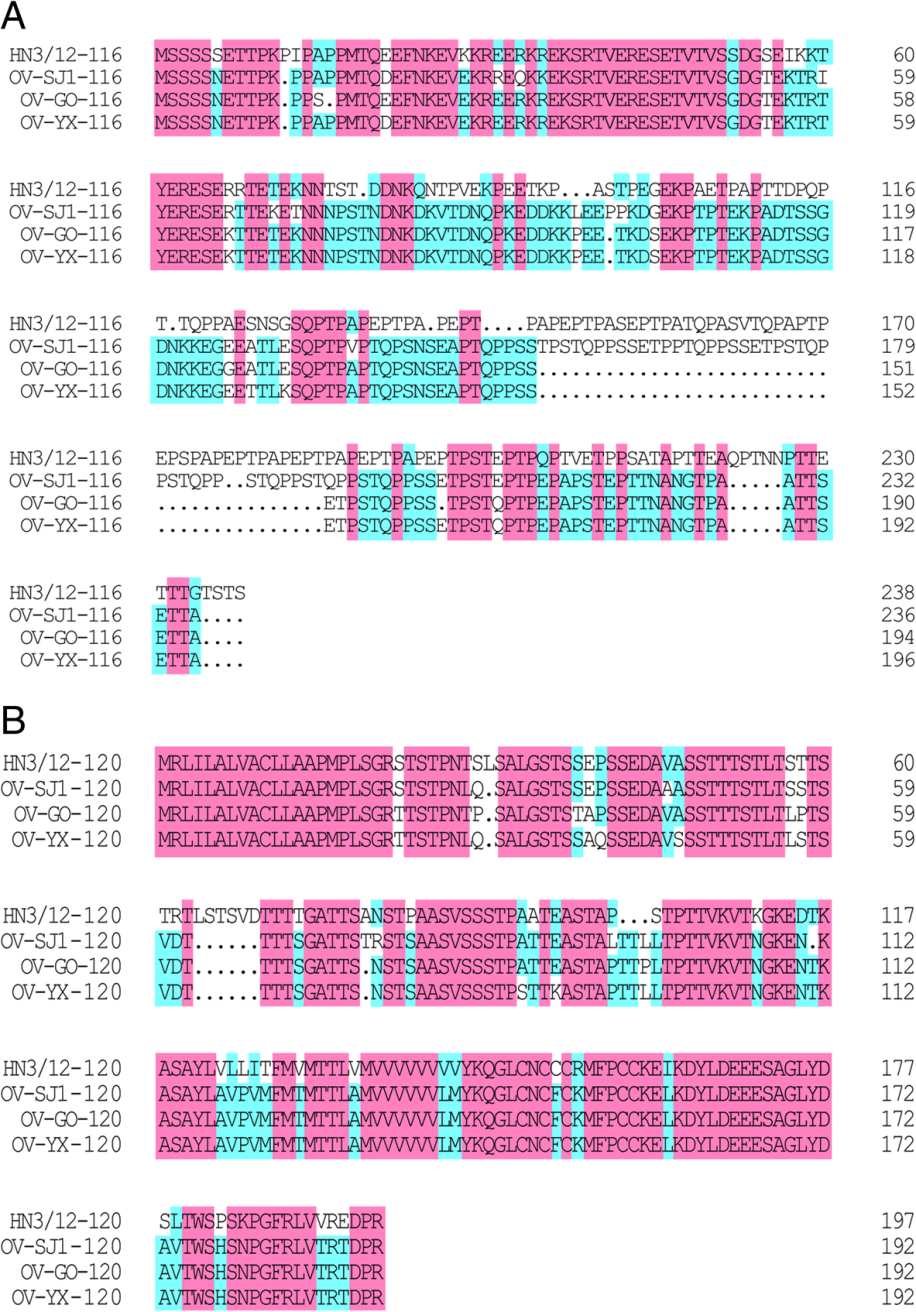
Amino acid sequence alignment of ORF116 and ORF120 coded by four ORFVs. Multiple alignment was performed by Clustal Omega (available at http://www.ebi.ac.uk/Tools/msa/clustalo/) and DNAMAN software. Four ORFV strain accession numbers are shown in Table 2. The *pink box* indicates 100% ID of amino acid sequence among five ORFV strains. The *cyan box* indicates >50% ID of amino acid sequence among five ORFV strains

ORFV-HN3/12 open reading frames were aligned with those of the OV-GO, OV-YX, OV-NP and OV-SJ1 strains under the revision suggested by Chi et al. [15]. Pairwise protein alignments revealed that the HN3/12 strain shared 110 genes, 10 genes, 7 genes and 5 genes with greater than or equal to 90%, 80–89%, 60–79% and <60% amino acid identity, respectively, with OV-GO. Compared with OV-YX, HN3/12 shared 114 genes, 11 genes, 6 genes and one gene with greater than or equal to 90%, 80–89%, 60–79% and <60% the identity of amino acid sequences. With the OV-SJ1 strain, the Henan strain shared 112genes, 11 genes, 4 genes and two genes. HN3/12 shared 111 genes, 7 genes, 2 genes and four genes with OV-NP (Table 3). Multiple amino acid sequence alignments were performed based on four amino acid sequences (001, 005, 116 and 120) from ORFV strains. The open reading frame identities of the pairwise amino acid sequence alignment were <80%. The results of multiple alignments revealed that the homology of ORF001 (72.00%) was higher than ORF005 (61.34%) (Fig. 4), when HN3/12 was compared with four strains from Fujian province. In a comparison of HN3/12 with OV-GO, OV-YX and OV-SJ1 strains, the identity value of ORF120 (71.34%) was greater than ORF116 (55.06%) (Fig. 5). Additionally, the identities of ORF001 and 005 amino acid sequences among the four Fujian ORFV strains are greater than HN3/12. ORF116 and 120 have a similar pattern.

**Table 3:** ORFs of HN3/12compared with the corresponding ORFV strains GO, YX, SJ1, NP

HN3/12	% Id (aa)							
	> = 90		80–90		60–79		<=60	
	Numbers	ORFs	Numbers	ORFs	Numbers	ORFs	Numbers	ORFs
GO	110	002,007,009…	10	012,013,061,080,104,113,115,118,119,121	7	001,005,059,102,112,120,134	5	103,109,110,116,132
YX	114	007,008,010…	11	002,012,061,080,110,112,113,115,118,119,121	6	001,005,109,120,132,134	1	116
SJ1	112	023,053,055…	11	001,012,013,024,061,080,102,109,113,121,134	4	005,103,120,112	2	116,132
NP	111	023,026,087…	7	012,061,080,104,109,121	2	001,102	4	002,005,103,132

## Discussion

Orf, caused by ORFV, is a zoonotic disease that mainly affects sheep and goats globally, and has been listed as a class I animal disease in China [13]. However, little information about the epidemiology and distribution characteristics of this virus is available. Currently, only nine ORFV strains have been isolated and relatively completely sequenced: NZ2 in New Zealand, IA82 and SA00 in America, D1701 in Germany, NA1/11, OV-GO, OV-YX, OV-NP and OV-SJ1 in China [20–22, 24, 25]. No genomic information about ORFV virus has been reported from central China. Therefore, ORFV needs to be explored further in order to effectively prevent and control this disease.

In this study, we successfully isolated and identified a novel orf virus strain named HN3/12 from infected sheep in a village in Wuyang, in the Henan province of central China. The entire genomic sequence of ORFV-HN3/12 is about 136.6-kbp in length, with 63.67% G + C content, and contains 2.79-kbp inverted terminal repeats (ITRs). The features are in agreement with other previously reported ORFV strains, such as OV-GO, OV-YX, OV-NP, OV-SJ1, OV-NZ2, OV-SA00, OV-IA82, OV-NA1/11, and other PPVs. The length of the ITR in HN3/12 strain is the second shortest among these ORFV strains.

Most published phylogenetic analyses and molecular epidemiology of orf virus have been investigated based on the highly conserved genes of *ORFV011 (B2L)*, *ORFV059 (F1 L)* and/or *ORFV020 (VIR)* [23, 26–28]. The sequences of *ORFV011* and *ORFV059*, which were regarded as epidemiologically relevant sequences, are usually used for phylogenetic analysis of orf virus [16, 19, 29]. *ORFV020 (VIR)* as a marker, and is also employed in molecular epidemiological studies of ORFV [30, 31].

Phylogenetic analysis of the conserved regions (*011 and 059* genes) indicated that ORFV-HN3/12 is closely related to ORFV-NA1/11, a sequenced orf virus strain isolated from the Jilin province, in the northeast of China [22]. Phylogenetic analysis of the *ORFV011* gene showed that the HN3/12 isolate clusters together with ORFV strains from northern China. However, the strain is closest to the ORFVs coming from the Fujian province, according to the analysis of *ORFV059* gene. This suggests that phylogenetic analysis based on single gene may have some bias.

The results of the phylogenetic analysis based on the genomic sequences of 13 PPV strains revealed that HN3/12 has a closer relationship with NA1/11 than to the others, and this is consistent with the phylogenetic analyses of the *011* and *059* genes. The two virus strains originated in sheep and were isolated from the north of China (NA1/11 from Jilin province and HN3/12 from Henan province), suggesting that they may stem from the same origin. This implies that the strain can be utilized for designing a vaccine to reduce outbreaks of orf in sheep herds at these areas.

Furthermore, 132 amino acid sequences of HN3/12 were compared with that of the four Fujian strains. The results of pairwise sequence alignment showed that the identity of most ORFs are more than 90%, based on protein sequence analysis, and some ORFs are less than 80% (Table 3 and Additional file 1: Table S1). Among the ORFs with low identity, four ORFs (001, 005, 116 and 120) from five ORFV strains were analyzed further based on multiple amino acid sequence alignment. The homology of ORF001 and 120 is greater than the homology of 005 and 116 . Moreover, based on ORF001 and ORF005 sequence alignment analysis, the four Fujian strains share higher identity with each other, while HN3/12 shares a lower identity with the Fujian strains (Fig. 4). The identities of ORF116 and 120 in the Fujian strains, except for OV-NP (there is a deletion of ORF114–120 in OV-NP), are higher than with the HN3/12 strain (Fig. 5). This may be attributed to their having the same origin and fewer mutations between them. In these ORFs with low identity, *ORFV109* and *110* genes encode type II EEV glycoproteins, which are expressed in inter- and extra-cellular enveloped virions [21]. ORFV132 protein is a VEGF-like protein and plays a crucial role in the development of lesions induced by ORFV [32] (Additional file 1: Table S1). However, the functions of some proteins, such as ORFV001, 005, 116, 120, are still unknown, and require further exploration.

## Conclusions

In summary, the isolated HN3/12 virus is a novel ORFV strain based on clinical manifestations, PCR amplification of *ORFV011*and *059*, and genome sequences. New genomic information of this ORFV strain from Wuyang country, in central China’s Henan province, was obtained, which provides basic knowledge about ORFV to allow taking reasonable precautions and to control the disease. This may assist other researchers to explore the molecular epidemiology and the diversity of genomic sequences of ORFV. Future studies will reveal more relevant knowledge about ORFV and isolate more strains from different regions in China or worldwide, for a deeper, more comprehensive understanding of ORFV biology and epidemiology.

## Methods

### Sheep herds and tissue collection

The case described presently originated on a farm keeping about 400 sheep located in a village in Wuyang, Henan province, in the north-central region of China(113.6 E, 33.43 N), in January, 2012. Eleven lambs, aged one to six months, and six ewes presented with typical skin lesions on the muzzle, lips, and teats, possibly due to an Orf virus infection. The afflicted animals were cured within 5 weeks and suffered no deaths. Skin tissue samples with gross pathologic changes and scabs were collected, using a pair of sterilized tweezers, from four lambs and two ewes and stored at −80 °C for virus isolation and further analyses. Sera from the corresponding animals were collected from the jugular vein and preserved at −80 °C for further testing. All animal procedures were reviewed and approved by the Institutional Animal Care and Use Committee at South China Agricultural University (the certification number: CNAS BL0011).

### Isolation and culture of Orf virus

The isolation of the virus was performed according to previous methods with some modifications [33, 34]. Briefly, tissue samples were mechanically homogenized with a disposable pestle (Sangon Biotech, Shanghai, China) in 1 × MEM medium (Hyclone, Logan, UT, USA) supplemented with gentamicin (50 μg/ml), penicillin (100 U/ml), and streptomycin (100 μg/ml), followed by centrifugation at 3000×g for 10 min. Clarified supernatant was passed through 0.45 μm filters and inoculated onto OFTu cells. The cells were incubated at 37 °C in 5% CO_2_. Cells were observed daily with an inverted microscope for monitoring the progression of virus-induced CPE. Infected cells were passaged thrice after CPE appeared. When 60%–80% CPE was observed, cells and medium were harvested and stored at −80 °C for further experiments. The virus was isolated from the OFTu cell culture medium and a single clone of the viral strain was isolated by a plaque assay, performed according to previous reports [6, 9]. Briefly, OFTu cells were infected with serial dilutions of virus from 10^−1^ to 10^−6^ for 1 h at 37 °C in a 5% CO_2_ incubator. 3 ml MEM containing 5% FBS and 0.5% low melting point agarose (Sea KemW GTGW, Lonza, Rockland, ME, USA) was added after removing the old medium. Plaques caused by a single virus were visualized and picked at 4 or 5 dpi. Each isolate was acquired with a minimum of 2 or 3 plaques.

### Polymerase chain reaction (PCR) and gene sequencing

Total DNA was extracted directly from tissue suspensions (200ul) of the lip lesions or purified virion materials using a QIAamp DNA blood kit (QIAGEN, Duesseldorf, Germany), according to the manufacturer’s instructions. PCR of the *ORFV011* and *ORFV059* genes was performed. Two sets of primers were designed based on the OV-NZ2 genomic sequence to amplify these two highly conserved genes [20]. Primer sets were as follows: NZ2-ORFV011Fw: 5′- ACACCTTTCCCCAGAACCCCA-3′; NZ2-ORFV011Rv:5′- GTCCGAGCTCCAGTTGCTGACTT-3′; NZ2-ORFV059Fw: 5′- ACGTCATCACATGCGGGTCAGAG-3′; NZ2ORFV059Rv:5′- CTTCCTGTTCCTGGCGGGCAT-3′. PCR reactions were carried out in 50 μl reaction volumes, which contained 10 μl of 5 × PCR buffer (10 mMTris–HCl and 50 mMKCl), 2 μl of DNA template, 200 μMof each dNTP, 0.4 μM of each primer, 25 μM MgCl_2_ and 0.5 μl of Taq polymerase (Takara, Dalian, China). PCR reactions were performed in a thermocycler (GeneAmp PCR 2400, Perkin Elmer, Shelton, CT, USA) for 32 cycles of denaturation at 95 °C for 1 min, annealing at 56 °C for 30s and extension at 72 °C for 1 min, followed by a final extension at 72 °C for 10 min. The amplified products were resolved by 1% agarose gel electrophoresis and analyzed with an IS-1000 Digital Imaging System (Alpha Innotech Corp. San Leandro, CA, USA).

The PCR products were individually purified using a MiniElute gel extraction kit (QIAGEN, Duesseldorf, Germany), according to manufacturer’s procedures. Purified gene fragments were cloned into pMD-19 T vector (Takara, Dalian, China) and transformed into *E.coli* Top10 competent cells. The existence of the insert was determined by restriction endonuclease (EcoRI and BamHI) digestion and agarose gel electrophoresis. Two or three positive clones for each gene were sent to BGI-Shenzhen for sequencing.

### Genomic DNA extraction and sequencing

Infected cells were harvested when 80% CPE was observed by scraping the bottom of the tissue culture flasks. The mature virions were purified from the infected cells by sucrose gradient ultra-centrifugation as previously described [9, 16]. Viral genomic DNA was extracted from tissue purified virion material using a Virus DNA purification Kit (Roche, Basel, Switzerland), following the manufacturer’s instructions. This genomic DNA preparation was sequenced using the Paired-End method on a high-throughput Illumina system [35, 36]. Briefly, viral genomic DNA was interrupted randomly by sonication to produce a series DNA fragments with sizes less than or equal to 800 bp. The DNA fragments were then repaired and adapters ligated to both ends. Sequence data were assembled with the Phrap and CAP3 software programs. Gaps were closed by primer walking and verified by sequencing of PCR products.

Genomic DNA composition, structure, repeats, and restriction enzyme patterns were analyzed using the Genetics Computer Group (GCG) version 10 software package. Open reading frames (ORFs) longer than 30 codons were evaluated for coding potential and ORFs greater than 60 codons were subjected to homology searches with BLAST in the NCBI database (https://blast.ncbi.nlm.nih.gov/Blast.cgi). In addition, the Hexamer (ftp.sanger.ac.uk/pub/rd) and Glimmer programs were used to evaluate coding potential. Based on these criteria, 132 ORFV putative genes were annotated and orthologous ORFs were similarly numbered. The genome sequence was submitted to GenBank with the Accession Number: HN3/12: KY053526.

### Sequences alignment and phylogenetic analysis

The nucleotide and deduced amino acid sequences of the HN3/12 isolate were aligned with Sequencer version 3.0 (Gene Codes Corp., Ann Arbor, MI, USA) and compared to sequences of the corresponding genes of those of PPVs available in the GenBank database using the online BLAST tool (https://blast.ncbi.nlm.nih.gov/Blast.cgi). Two phylogenetic trees of*ORFV011* and *059* gene sequences were constructed by the neighbor-joining method, with 1000 bootstrap replicates, using MEGA version 5.2. Phylogenetic comparisons were done based on the whole genomic nucleotide sequences, including terminal repetition, of PPV strains with MEGA 5.2 software using the maximum-likelihood method. The 13 PPVs genomic sequences were deposited into GenBank with the following Accession Numbers: HN3/12: KY053526, OV-YX: KP010353; OV-GO: KP010354; OV-NP: KP010355; OV-SJ1: KP010356; IA82: AY386263; SA00: AY386264; NZ2: DQ184476and F00.120R: GQ329669; VR634: GQ329670; BV-AR02: AY386265.

### Amino acid sequence alignment of ORFVs

Pairwise sequence alignments of 132 ORFs from the HN3/12 strain with eight ORFV strains (OV-GO, OV-YX, OV-NP, OV-SJ1, NA1/11, IA82, NZ2 and SA00) were performed using EMBOSS Needle in the EMBL-EBI database (available at http://www.ebi.ac.uk/Tools/psa/emboss_needle/). Multiple amino acid alignment for ORF001, ORF005, ORF116 and ORF120 from the Fujian ORFV strains and the Henan strain were performed by Clustal Omega (available at http://www.ebi.ac.uk/Tools/msa/clustalo/) and DNAMAN software.

## Additional file

Additional file 1: Table S1 ORFs of HN3/12 compared with the corresponding of ORFV strains YX, NP, GO, SJ1, NZ2, IA82, NA1/11 and SA00. (XLS 64 kb)

## Abbreviations

BLAST: Basic Local Alignment Search Tool
bp: Base pair
BPSV: Bovine popular stomatitis virus
CPE: Cytopathic effect
ITR: Inverted terminal repeat
NGS: Next Generation Sequencing
OFTu: Ovine fetal turbinate
ORF: Open reading frame
ORFV: Orf virus
PCPV: Pseudocowpox virus
PCR: Polymerase chain reaction
PPV: Parapoxvirus
PVNZ: Parapoxvirus of red deer in New Zealand
SPPV: Squirrelparapoxvirus
